# Assessment of risk factors and preventive measure compliance against COVID-19 among healthcare workers at tertiary care hospital: a retrospective study

**DOI:** 10.3205/dgkh000435

**Published:** 2023-04-28

**Authors:** Arifa Khatoon, Reema Aslam, Shabnam Bilal, Sadia Naz, Farhan Zaffar, Shahbaz Ahmed Khan, Rao Muhammad Ramzan, Saima Noreen, Kiran Phoolzaib, Zahra Batool, Kahsma Saleem, Saba Rasheed

**Affiliations:** 1 Infection Control Department, Indus Hospital & Health Network, Karachi, Pakistan

**Keywords:** COVID-19, health-care workers, risk category, personal protective equipment, infection prevention and control

## Abstract

**Introduction:**

SARS-CoV-2 has created a significant challenge to healthcare systems, since the disease has spread rapidly, outweighing hospital capacity and exposing Health Care Workers (HCWs) to the risk of infection. The main objective of this study shows the HCW’s self-reported use of Personal Protective Equipment (PPE), symptoms, and exposure to revealed and suspected people during the pandemic, as well as the implementation of infection prevention and control (IPC) guidelines that effectively limit the spread of the infection among healthcare personnel.

**Method:**

A single-center retrospective cohort study has been done at a tertiary care hospital. There were 3,651 hospital employees of these 1,890 HCWs and 1,761 nonclinical staff among those who were proven or suspected COVID-19 cases and had symptoms were included. The data was gathered using a standardized self-assessment questionnaire. Information about quarantine protocol and line listing was collected through telephonic conversations.

**Result:**

The majority of the participants were males (66%). The average age was 32.1±7.62. Out of 432 HCWs, 32.9% with positive SARS-CoV-2 PCR findings were nurses, 19.2% were doctors, and 47.9% were non-clinical employees from the hospital’s inpatient and outpatient departments. 31.5% had a higher-risk exposure, 64.1% had a moderate-risk exposure, and 4.4% of practitioners with COVID-19 had a lower-risk exposure. A statistically significant association was found between COVID-19 disease and adherence to PPE and risk exposure.

**Conclusion:**

This study represents the healthcare workers’ experience with COVID-19 patients in the early stages of the pandemic and emphasizes the measures required to overcome the problems, however, this study highlights that HCWs are being progressively infected with COVID-19 as a result of inadequate/ inappropriate PPE wear.

## Zusammenfassung

**Einleitung:**

SARS-CoV-2 stellt eine große Herausforderung für die Gesundheitssysteme dar, da sich das Virus rasch ausbreitet, die Kapazitäten der Krankenhäuser nicht ausreichen und das Gesundheitspersonal einem Infektionsrisiko ausgesetzt ist. Das Hauptziel dieser Studie war es, die von den Beschäftigten des Gesundheitswesens selbst angegebene Verwendung von persönlicher Schutzausrüstung (PSA), die Symptome und die Exposition gegenüber Personen mit nachgewiesener Infektion oder Infektionsverdacht während der Pandemie sowie die Umsetzung von Richtlinien zur Infektionsprävention aufzuzeigen.

**Methode:**

Es wurde eine retrospektive Kohortenstudie in einem Krankenhaus der Tertiärversorgung durchgeführt. An der Studie nahmen 3.651 Krankenhausmitarbeiter teil, darunter 1.890 Angestellte des Gesundheitswesens und 1.761 nicht-klinische Mitarbeiter, die nachweislich oder vermutlich an COVID-19 erkrankt waren und Symptome aufwiesen. Die Daten wurden mithilfe eines standardisierten Fragebogens zur Selbsteinschätzung erhoben. Informationen über das Quarantäneprotokoll und Erfassungslisten wurden in Telefongesprächen gesammelt.

**Ergebnisse:**

Die Mehrzahl der Teilnehmer waren Männer (66%). Das Durchschnittsalter betrug 32,1±7,62 Jahre. Von den 432 Beschäftigten mit positivem SARS-CoV-2-PCR-Befund waren 32,9% Krankenschwestern und -pfleger, 19,2% Ärzte und 47,9% nicht-klinische Mitarbeiter aus den stationären und ambulanten Abteilungen des Krankenhauses. 31,5% der Ärzte wiesen eine Exposition mit höherem Risiko auf, 64,1% eine Exposition mit mittlerem Risiko und 4,4% der an COVID-19 erkrankten Ärzte wiesen eine Exposition mit geringerem Risiko auf. Es konnte ein statistisch signifikanter Zusammenhang zwischen der Erkrankung an Covid-19 und der Einhaltung der PSA sowie der Risikoexposition nachgewiesen werden.

**Schlussfolgerung:**

Die Studie stellt die Erfahrungen des Gesundheitspersonals mit COVID-19-Patienten in der Anfangsphase der Pandemie dar und unterstreicht die Notwendigkeit der Maßnahmen, die zur Bewältigung der Probleme erforderlich sind. Die Studie zeigt jedoch auch, dass sich das Gesundheitspersonal aufgrund des Tragens unzureichender/ unangemessener PSA zunehmend mit COVID-19 infiziert.

## Introduction

The COVID-19 pandemic has already resulted in over 14 million infections and 600,000 fatalities worldwide [1]. Since its first public reporting on December 31, 2019, it has expanded to 216 nations in just a few months and is considered a continuing worldwide epidemic [2]. Pakistan’s neighbors, particularly China, were severely impacted, with the COVID-19 outbreak emerging for the first time [3]. The Ministry of Health, Government of Pakistan, reported the first case of COVID-19 in Pakistan on February 26, 2020, in Karachi, Sindh province [4].

The burden of COVID-19 has increased globally in terms of morbidity, mortality, and economic disaster [3], [5]. COVID-19 has provided a significant problem for healthcare systems since the disease has spread rapidly, outpacing hospital capacity and bringing HCWs at high risk of infection [4] due to direct contact with COVID-19-infected patients [6]. To minimize healthcare transmission and protect workers and vulnerable patients in healthcare settings, it is critical to categorize and manage HCWs who have been exposed to a patient with COVID-19 [7]. Infection prevention among HCWs is essential to minimize morbidity and possible death, maintain health system capacity, and prevent secondary transmission [8]. Based on recent experience with other respiratory viruses, PPE is suggested [9]. Reduce the risk of COVID-19 transmission in hospital settings by standardizing HCW procedures [1], limiting infection dissemination to and from HCWs [10]. HCWs were mandated by the Chinese government to diligently implement preventative measures and enhance protective measures against droplet isolation, contact isolation, and air isolation. Infection prevention and control IPC measures suggested by the WHO for preventing and reducing transference include hand hygiene, medical masks, and personal protective equipment PPE [7]. There is evidence that taking correct precautions during outbreak management can drastically alter the outbreak’s trajectory [3]. It’s also essential to improve HCWs’ and the community’s understanding and preventative practices concerning COVID-19 by providing updated information [7]. There is noticeable evidence showing that proper measures during outbreak management could remarkably change the course of the outbreak [2]. Also, improving the knowledge and prevention practice of HCWs and the community through regular updates about COVID-19 is crucial [7].

This research assessed HCWs’ self-reported compliance with PPE, symptoms, and exposure to confirmed and suspected people during the pandemic, as well as IPC implementations, which can substantially restrict viral transmission and are fundamental to healthcare personnel’s safety. The goal of this study is to identify the frequency of emergent coronavirus outbreaks, which have highlighted concerns regarding nosocomial transmission, or transmission within hospital settings. According to IPC, healthcare practitioners’ failure to follow suggested personal protective behaviors is a significant source of transmission. This brief evidence assessment focused on the existing literature on emerging infectious disease outbreaks to see whether characteristics were linked to healthcare workers’ compliance with behavioral and social infection control strategies. Moreover, personnel working in healthcare facilities are known to be more vulnerable to infectious disease agents such as COVID-19, but, they may also have improved infectious disease preventive measures than those in other occupations, effectively reducing disease transmission. In addition, we investigated the causes of HCW infection and ways of overcoming the problem.

## Method

### Study design and setting

**Figure 1:**
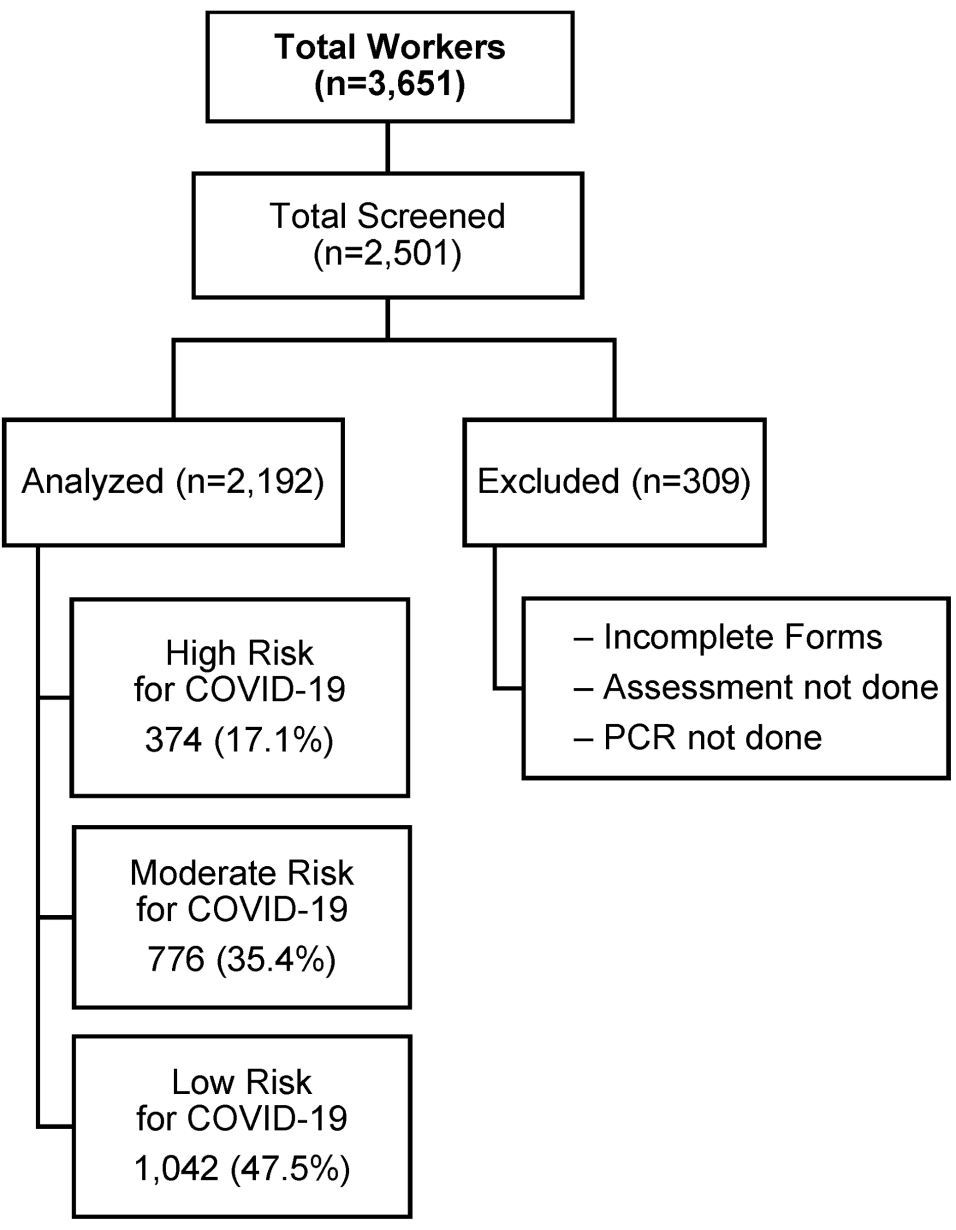
Figure1: Retrospective cohort study flow diagram [16]

A single-center retrospective cohort study has been done at a tertiary care hospital among HCWs and nonclinical staff in Karachi, Pakistan that operates a 300-bed inpatient facility and 35-bed emergency department. The flow diagram of this study is depicted in Figure 1 (Fig. 1).

### Data collection

The data was gathered using a standardized self-assessment questionnaire. The participants who were proven or suspected COVID-19 cases, as well as who had symptoms, were requested to submit questionnaires to the ICD after doing their COVID test according to protocol. Incomplete questionnaire data were eliminated from the study because of details of incomplete information. This pandemic has imposed enormous pressure and stress on hospital staff. Initially, it was very panic to manage and overcome the circumstances to identify the COVID-19 symptoms as it was attributed to shifting knowledge regarding COVID -19 from the international agencies. 

**Figure 2:**
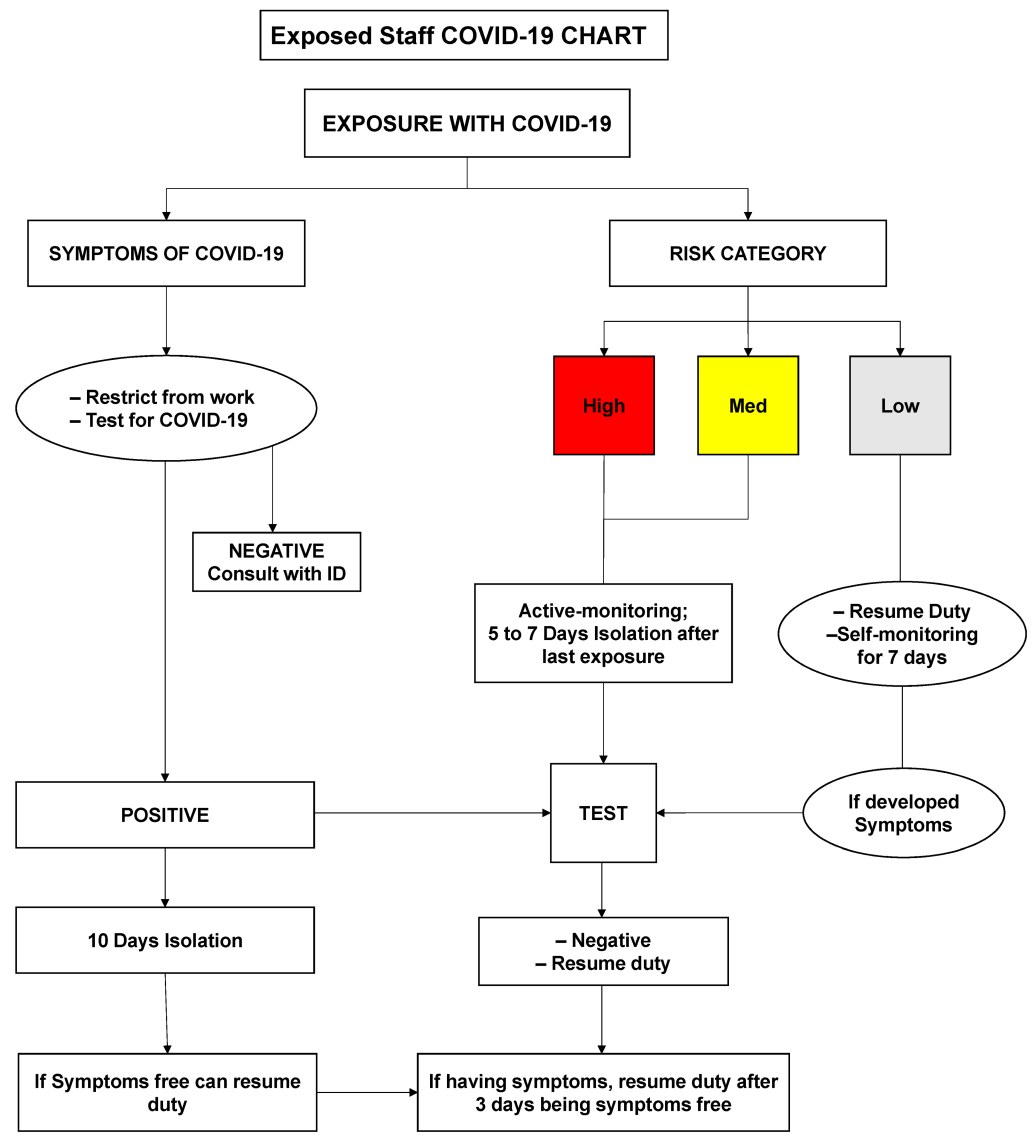
Exposure of COVID-19 Flow Diagram [1]

The questionnaire was designed by the hospital’s recommendations sent to impacted personnel, which were compatible with the “IPC during health care when COVID-19 infection is suspected. Intervening guidance” proposed by WHO and CDC. The standardized self-reported IPC measures suggested by HCWs recommendation were early recognition and immediate placement, hand hygiene, distancing, use of PPE based on risk, and contact and airborne precautions for suspected COVID-19. Assisting HCWs who have been exposed to a person who has COVID-19, whether suspected or proven. The infection control practitioner analyses the risk of exposed healthcare workers. The following characteristics were used to determine the severity of the infection increase:

1. health-care employees’ exposure to proven and suspected patients or coworkers;
2. risk categorization, which includes high risk, moderate risk, and low risk (Figure 2 (Fig. 2));
3. close contact with one or both of them not wearing masks and having symptoms;
4. no close contact with both of them wearing masks if the respondents were exposed to confirmed and suspected patients (defined as flu-like symptoms with fever, sore throat, body ache, headache, diarrhea, smell & tasteless or cough).

### Data analysis

Data were analyzed using statistical software SPSS (SPSS 25.0; SPSS Inc, Chicago, IL, USA, IBM). Frequencies and percentages were reported for all categorical variables while means (standard deviations) and median (interquartile range) were reported for all continuous variables as descriptive statistics and compared using the chi-square test or Pearson’s exact test was used to find out the association between PCR and PPE compliance among COVID patients. A p-value <0.05 was considered statistically significant.

## Results

The total number of participants was 2,501. Analysis was performed on 2,192 of them since data for 309 participants were missing, and the self-reported information was incomplete. The majority of the participants were males (66%) and (34%) were females. Clinical and demographic variables detailed were depicted in Table 1 (Tab. 1). The average age for males and females was 32.10±7.62. Out of these participants, 72.4% were exposed to a confirmed COVID-19 carrier, compared to 27.5% who were suspects. The percentage of nurses, doctors, and other non-clinical staff who were in contact was 32.9%, 19.2%, and 47.9% respectively. Only 23.6% of participants showed symptoms, whereas 76.4% were symptom-free. In contrast, risk assessment is divided into three categories: high, moderate, and low. There is a significant association between risk of exposure and COVID-19 findings (P-value <0.001) among healthcare professionals depicted in Table 2 (Tab. 2). Prevalence of infection was highest among those not following (64%) PPE guidelines followed by who follow PPE guidelines and have a lower chance of exposure (63%).

Among the positive findings of Covid-19, there is a significant association with PPE compliance (P-value <0.001). Only 40.3% who showed compliance with PPE tested positive, compared to 59.7% of those who did not (Table 3 (Tab. 3)). Noncompliance with facemask usage in non-clinical shared workplaces (e.g. break time, doctor close container/rooms, or aggravation) or during activities such as meals or tea time when facemasks were removed, and social distancing was not maintained were common in these higher-risk exposures. PPE compliance differs significantly from the type of health care personnel (P-value=0.001). Doctors have the lowest compliance rate (61.8%), while nurses have the highest (77.5%).

## Discussion

The study was conducted to assess the effectiveness of infection prevention and control strategies among HCWs, to analyze potential risk factors and clinical presentation of SARS-CoV-2 infection [11] after administering the WHO COVID-19 contact risk assessment questionnaire, used worldwide for healthcare worker exposure risk evaluation and isolation, which has contributed to a reduction in HCW exposure among other HCWs was adapted by our institution. Previous research has also highlighted COVID-19 IPC difficulties in hospital settings, underlining the issues of personal safety of healthcare personnel.

Our study shows that the most higher-risk exposures staff were exposed to suspected with non-systematic colleagues in areas where there was assumed to be no risk of COVID-19 exposure, such as physician duty rooms, common rooms, changing rooms, and cafeterias where people gathered for lunch or tea breaks and shared their plates and food with buddies as is considered the norm, and non-compliance with PPE was also observed because health care workers and other non-clinical area staff assume that there is no risk of COVID-19 exposure. Resulting, among HCWs who shared the same space (break time, doctor closing container/rooms, or feeling relieved after removing the mask in front of friends and colleagues), the positive ratio is high. Results from the U.S. [12] and Iran [9] reported that 25%, and 11.4% of healthcare staff testing positive, respectively, had a significant risk of exposure to the workplace and viral dissemination among frontline HCWs [13], [14], [15] which is consistent with our findings.

On the other hand, the Ethiopian study focused on the occurrence and practice of HCWs about COVID-19 and revealed risk categories related to HCWs’ COVID-19 infection control measures. HCWs faced significant problems during viral outbreaks since they are on the front line of prophylaxis and had the highest chance of contracting the illness and disseminating it to their families and communities [7]. They also experience exposure among HCWs, as well as concerns comparable to those we’ve seen, such as all high-risk exposure occurring in non-clinical settings such as nursing stations and break rooms, and exposure without a mask during meals when it has to be removed.

## Conclusion

The primary line of protection against COVID-19 infection is the healthcare personnel. However, preliminary research suggests that HCWs are being progressively infected with COVID-19 as a result of inadequate/inappropriate PPE wear. Along with all of this, most HCWs have lunch or tea with their coworkers, which raises the chances of infection cross-transmission. Resulting, it is advised that authorities emphasize HCW protection by education, training, and sensitization of staff to implement the COVID-19 SOPs to avert epidemic situations in institutions and communities.

To deal with this pandemic, the government implemented strategic planning and early management, and healthcare settings also combated the issue to undertake preventative measures for HCWs. As a result, government and institutional level early preparedness is ineffective until all individuals take responsibility to follow COVID-19 SOPs. We can prevent our community by emphasizing the importance of education and training programs for healthcare workers to control and prevent COVID-19 infection. Vaccination is another powerful method for disease prevention and control [14].

## Notes

### Competing interests

The authors declare that they have no competing interests.

### Ethical Approval 

The study was conducted after obtaining an Institutional Board review (IRB) under number IRD_IRB_2021_01_019 at The Indus Hospital.

## Figures and Tables

**Table 1:**
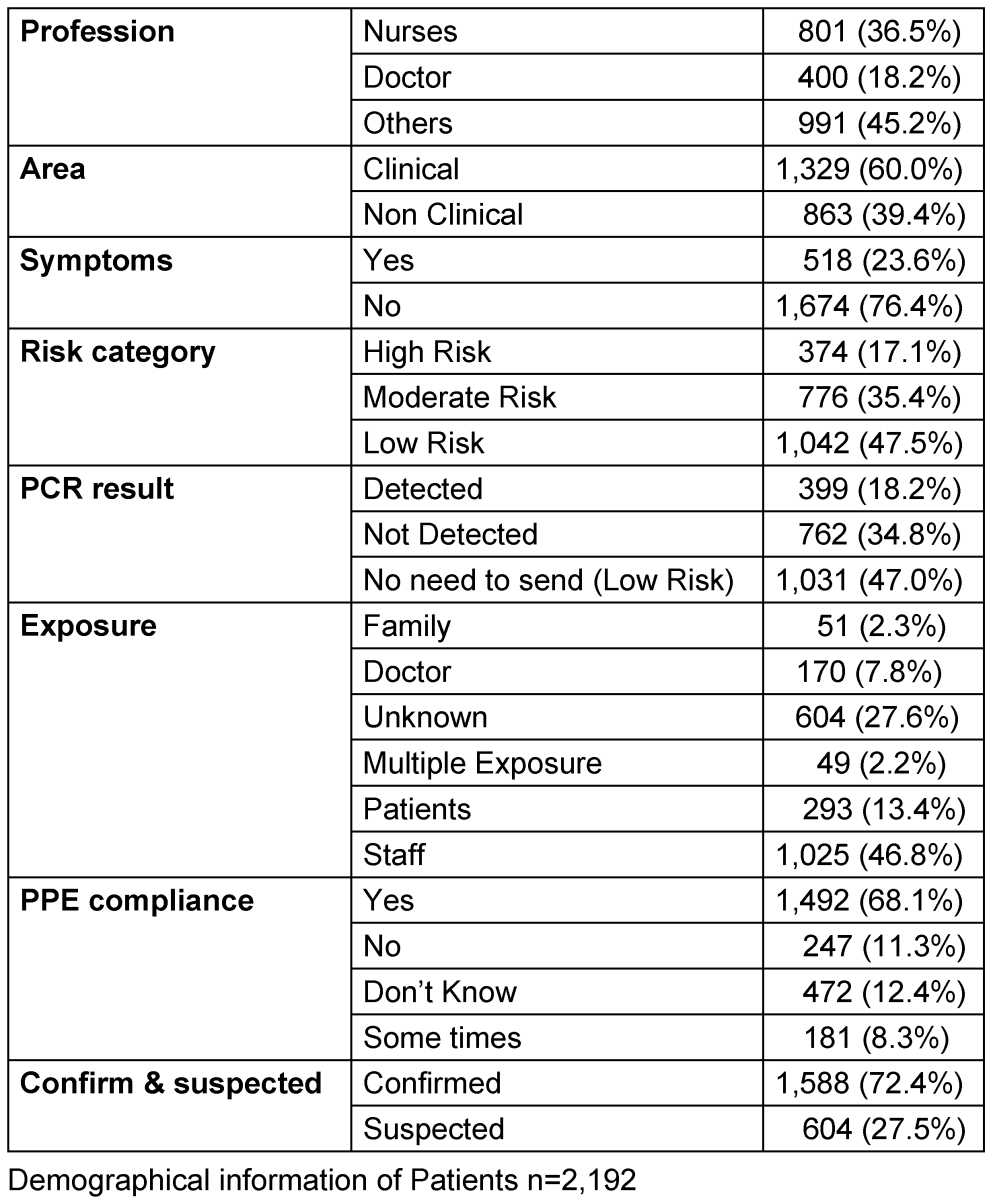
Clinical and Demographic variables

**Table 2:**
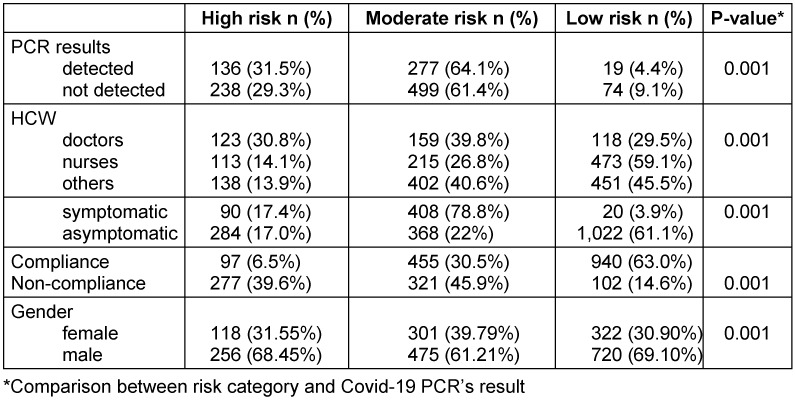
Comparison between risk category and Covid-19 PCR’s result

**Table 3:**
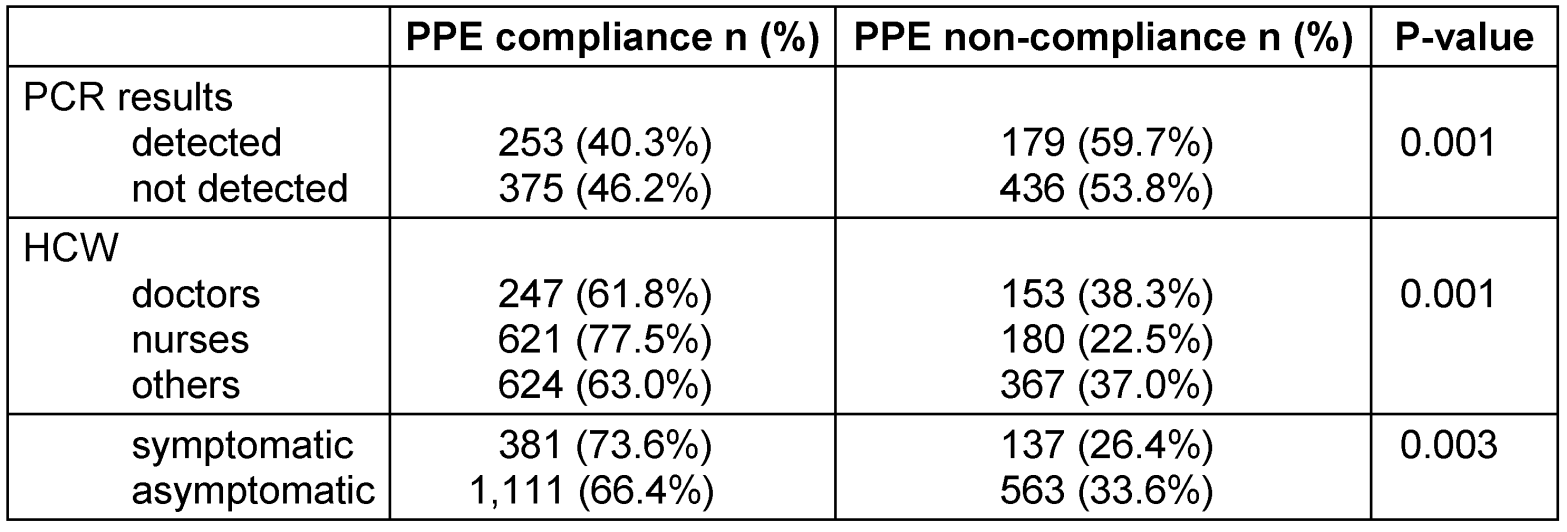
Comparison of adherence to PPE compliance and non-compliance
